# Virtual Screening in Search for a Chemical Probe for Angiotensin-Converting Enzyme 2 (ACE2)

**DOI:** 10.3390/molecules26247584

**Published:** 2021-12-14

**Authors:** Iryna O. Kravets, Dmytro V. Dudenko, Alexander E. Pashenko, Tatiana A. Borisova, Ganna M. Tolstanova, Sergey V. Ryabukhin, Dmitriy M. Volochnyuk

**Affiliations:** 1 Institute of High Technologies, Taras Shevchenko National University of Kyiv, 60 Volodymyrska Street, 01033 Kyiv, Ukraine; i.kravets@chem-space.com (I.O.K.); alev.pashenko@gmail.com (A.E.P.); gtolstanova@gmail.com (G.M.T.); d.volochnyuk@gmail.com (D.M.V.); 2 Chemspace LLC, 85 Chervonotkatska Street, Suite 1, 02094 Kyiv, Ukraine; 3 Enamine Ltd., 78 Chervonotkatska Street, 02660 Kyiv, Ukraine; d.dudenko@mail.enamine.net; 4 Institute of Organic Chemistry, National Academy of Sciences of Ukraine, Murmanska Street 5, 03028 Kyiv, Ukraine; 5 Palladin Institute of Biochemistry of the National Academy of Sciences of Ukraine, Leontovycha St. 9, 02000 Kyiv, Ukraine; tatianabiochem@gmail.com

**Keywords:** virtual screening, drug discovery, molecular docking, angiotensin-converting enzyme 2, ACE2, SARS-CoV-2, molecular probe, inhibitors, REAL database

## Abstract

We elaborate new models for ACE and ACE2 receptors with an excellent prediction power compared to previous models. We propose promising workflows for working with huge compound collections, thereby enabling us to discover optimized protocols for virtual screening management. The efficacy of elaborated roadmaps is demonstrated through the cost-effective molecular docking of 1.4 billion compounds. Savings of up to 10-fold in CPU time are demonstrated. These developments allowed us to evaluate ACE2/ACE selectivity in silico, which is a crucial checkpoint for developing chemical probes for ACE2.

## 1. Introduction

On 11 March 2020 the World Health Organization (WHO) declared a state of emergency to help contain the spread of COVID-19 [1]. Since December 2019, when the first SARS-CoV-2 cases were reported, the global number of cases has reached 223,296,909, and global deaths have reached 4,608,047 (as of 10 September 2021) [2].

Despite intensive studies, the biological mechanism behind the multisystem damage caused by SARS-CoV-2 infection remains unclear. One of the hypotheses is that the role of virus-induced ACE2 receptor downregulation is the key to understanding its pathogenesis. Before the COVID-19 outbreak, ACE2 was considered as a target mostly for treating ulcerative colitis [3], although with a low therapeutic value (due to possible severe side effects) [4]. It is known that SARS-CoV-2 enters a cell via the binding of the viral S-protein to the extracellular domains of the transmembrane ACE2, which leads to subsequent knockdown of surface ACE2 expression [5]. Cell entry by coronaviruses depends on binding of the viral spike (S) proteins to cellular receptors and on S protein priming by host cell proteases [5].

The investigation of the current biological consequences of inhibiting the catalytic activity of ACE2 is currently complicated due to the very limited data on small organic molecules’ binding to ACE2. This work aims to find the most promising molecule-candidates that fit the modern chemical probe criteria for ACE2-receptor. For this purpose, we applied in silico virtual screening in conjunction with molecular docking and molecular dynamics approaches to commercially available collections (e.g., the world’s largest collection of screening compounds for biological screening, the Enamine Screening Collection, comprising 3.2 million compounds [6]) and to a “make-on-demand” database (Enamine REAL database, 1.4 billion compounds [7,8]).

One of the key criteria for defining a compound as a chemical probe is its selectivity against the specific molecular target [9]. ACE2 is a metalloprotease that functions as a carboxypeptidase. ACE2 consists of 805 amino acid residues and has a transmembrane domain and a single HEXXH sequence that binds zinc (amino acid residue positions 374–378) [10]. ACE2, on the one hand, is an enzyme that has the functions described above and has a site of functional activity, and on the other hand is a place of attachment for the S-spike of SARS-CoV-2. These sites do not coincide in their location in the protein. To simulate the blocking effects of ACE2 as an enzyme, it is necessary to dock its active site. Thus, the X-ray structure with a known inhibitor in the enzyme’s active site is the best model for docking.

ACE2 is a membrane protein with an enzymatic domain located on the outer surface of human cells. It was so named because it was initially identified as a homologue (or version) of angiotensin-converting enzyme (ACE). This enzyme mediates the formation of a peptide hormone, angiotensin II, from angiotensin I. ACE has been widely studied and is well known for causing the vasoconstrictive effect (i.e., it causes muscle contraction in the vessel wall and narrows its lumen) [11]. Both ACE2 and ACE are part of the renin-angiotensin system (RAS), which regulates blood pressure [12]. Moreover, the catalytic domain of ACE2 is 42% identical to that of ACE. Although ACE2 is a close homologue of ACE, its catalytic activity is not blocked by drugs that inhibit ACE. ACE acts in opposition to ACE2, as an Ang II-forming enzyme versus the ACE2-Ang II-degrading mechanism. Thus, ACE2 catalyzes the conversion of angiotensin II to angiotensin 1–7, thereby counterbalancing ACE activity. We aimed to find only highly selective inhibitors of ACE2, because they can have contra functions. If both proteins are blocked, we will not be able to obtain the SARS-CoV-2 effect model. Therefore, in our study, we defined ACE as an “anti-target” for reasons related to selectivity [10].

## 2. Results and Discussion

### 2.1. Comparison of the Binding Sites

After a thorough inspection of the binding sites in ACE2 and ACE, we have identified specific structural differences which can be exploited for the sake of selectivity and specificity [13]. ACE2 and ACE have a number of similar fragments and segments, i.e., both are zinc-containing enzymes that are sensitive to anion activation [14,15,16]. However, unlike ACE, ACE2 acts as a carboxypeptidase and is not susceptible to inhibition by classical ACE inhibitors [13].

The comparison of protein–ligand binding sites provides valuable insights into yet-unexplored site similarities. Various stages of computational and chemical biology research can benefit from this knowledge. The search for putative off-targets and the establishment of polypharmacological effects by comparing binding sites has led to promising results for numerous projects [17]. Although binding site comparisons can now be retrieved from elaborate similarity databases [18], it is often advisable to perform additional comparisons, and this may even be necessary if proprietary structures are used [19]. Binding site comparisons can be applied to investigate minor dissimilarities between evolutionarily-related binding sites, as well as to reveal similarities between proteins that share no obvious global (sequence or structural) likeness.

A thorough inspection of the active sites revealed two pairs of fragments lying in opposite regions and playing a key role in the proteins’ binding properties: the amino acid residue TYR510 in ACE2 versus VAL518 in ACE and, most importantly, ARG273 vs. GLN281. Thirteen of these active site residues are conserved in the ACE2 and ACE enzymes: GLU145/GLU162, CYS344/CYS352, HIS345/HIS353, CYS361/CYS370, ASP368/ASP377, HIS374/HIS383, GLU375/GLU384, HIS378/HIS387, GLU402/GLU411, PHE504/PHE512, HIS505/HIS513, ARG514/ARG522 and TYR515/TYR523.

**Figure 1:**
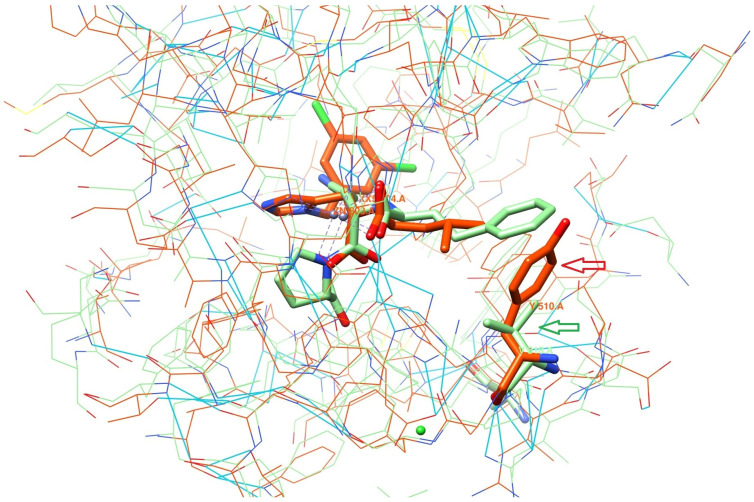
Comparison of ACE2 and ACE binding sites. In lime green—ACE with ligand lisinopril (PDB code: **1O86/LPR**), in orange—ACE2 with ligand ORE-1001 (PDB code: **1R4L/XX5**), in blue—hydrogen-bonding contacts. Lime green and orange arrows are pointing at VAL518 and TYR510 in ACE and ACE2, respectively.

These structural similarities result in the nearly identical way in which **XX5** and **LPR** are positioned in the superimposed active sites. TYR510 and THR347 line up the hydrophobic sub-pocket of ACE2 and allow this subsite to accommodate only small-to-medium-sized side chains, which is consistent with known substrate preferences. In the superposition of the ORE-1001-bound ACE2 and lisinopril-bound ACE structures, the TYR510 phenolic group of ACE2 occupies the space where the phenylpropyl group of the lisinopril is located (Figure 1).

**Figure 2:**
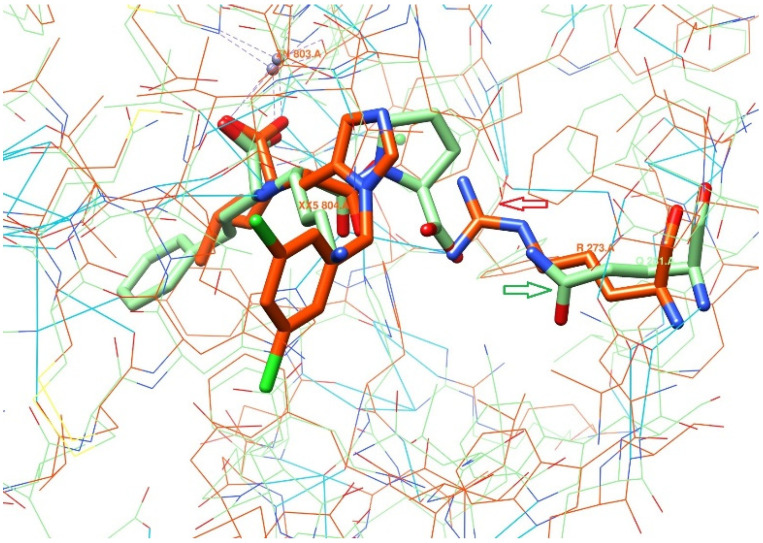
Comparison of ACE2 and ACE binding sites. In lime green—ACE with ligand lisinopril (PDB code: **1O86/LPR**), in orange—ACE2 with ligand ORE-1001 (PDB code: **1R4L/XX5**), in blue—hydrogen-bonding contacts. Lime green and orange arrows are pointing at GLN281 and ARG273 in ACE and ACE2, respectively.

Another essential difference between ACE2 and ACE is the substitution of ARG273 over GLN281 (Figure 2). The guanidinium group of ARG273 forms a salt bridge with the terminal carboxylic group of ORE-1001. Notably, the hypothesis about ARG273 being responsible for ligands’ binding to ACE2 has been proven experimentally by replacing arginine with a glutamine residue (R273Q mutation) using site-directed mutagenesis, which immediately resulted in the loss of affinity of the known inhibitors towards the receptor [13].

This crucial difference between the abovementioned residues in the homologically similar enzymes helps to explain why the potent ACE inhibitors, i.e., lisinopril, enalaprilat and captopril, are inactive against ACE2 [20]. The interaction identified above between the key residues in the active sites of ACE2 and ACE clearly plays a significant role in the observed discrepancy in substrate specificity and inhibitor binding profiles for these homologous enzymes.

### 2.2. Docking Model Preparation

An X-ray structure of the target protein was selected and prepared for docking according to the following criteria: good-quality resolution (1.5–2.5 Å), the presence of an active ligand in the crystal, organism: *Homo sapiens*, the date of publication and the journal ranking. Protein preparation started with the conversion of the protein into an ICM object. Here, an ICM object is the specific “internal” name of the molecular object (used only in MOLSOFT software, San Diego, CA, USA), which is usually formed after the preparation of the protein structure for docking. We removed all water molecules, then used the option “Optimize Hydrogens” to refine the orientation and charges of the HIS, PRO, ASN, GLN and CYS residues. The protein structure was prepared (all possible states were generated at pH = 7.4 ± 0.2) and minimized in the Merck molecular force field (MMFF) [21] (heavy atoms shift no more than 0.3 Å). Ultimately, we built docking models of ACE2 and ACE, and optimized them considering the following settings, explained in detail below.

“Grids” for ACE2 with the following restraints:

(1) First model:
  - ARG273—Interaction restraints: compulsory hydrogen bond;
  - The area near TYR510 is the positional/distance restraint of a spherical shape with a radius of 2 Å;
  - Interaction of ionic nature with Zn^2+^ metal.
(2) Second model:
  - ARG273—two positional/distance restraints—two spheres with a radius of 1 Å aiming to mark the positions of the oxygen atoms in the counter-ion carboxylic group;
  - The area near TYR510 is the positional/distance restraint of a spherical shape with a radius of 2 Å;
  - Ionic interaction with Zn2 + metal.
    “Grids” for ACE protein have the following restraints:
  - Interaction with Zn^2+^ metal;
  - LYS511—compulsory hydrogen bond.

**Figure 3:**
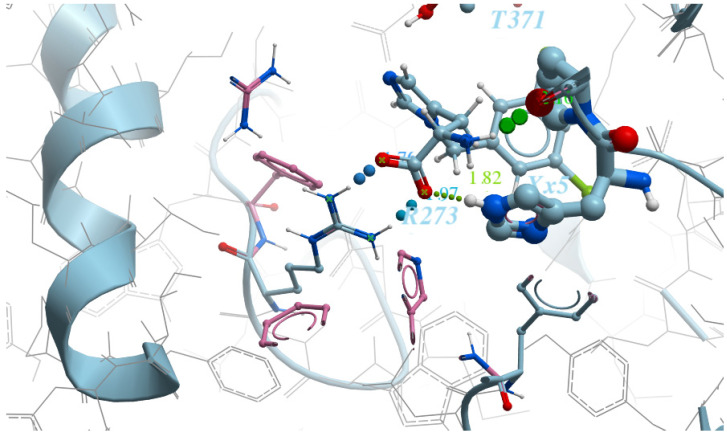
A salt bridge between ARG273 and the carboxyl group (first model of the ACE2 “grid”).

**Figure 4:**
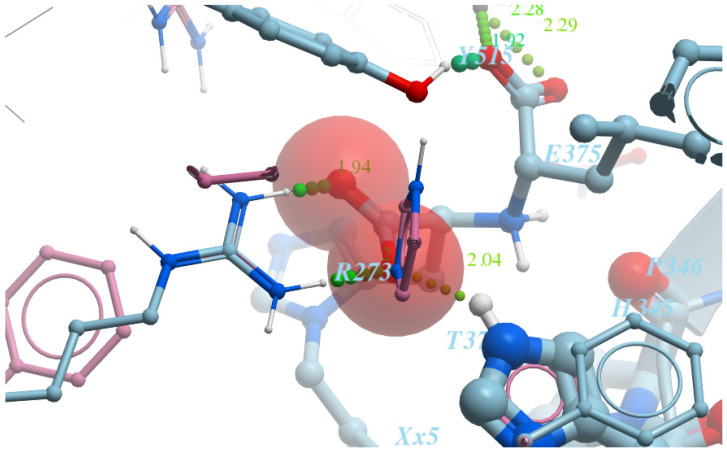
A distance restraint applied by means of two spheres with a radius of 1 Å, placed opposite the nitrogen atoms of ARG273 (second model of the ACE2 “grid”).

Detailed visualizations of the grids are presented in Figure 3 and Figure 4.

To test the docking models, all ligands from the ChEMBL database (https://www.ebi.ac.uk/chembl/, accessed on 21 July 2021) with experimentally measured activities—either Ki or IC50—were selected. There were 62 ligands in total (see Supplementary Materials, List S1).

In order to adjust our docking models, we used a free online service—the catalogue of useful decoys (DUD) [15]. For each known reference ligand, 100 property-matched “decoy”-molecules were selected, giving a total of 6200. All pre-built models were validated via in silico screening against the sets of reference inhibitors and the corresponding decoys. As a result, we obtained various sets to tune the grid models. Further, we examined their enrichment, usually by looking at a ROC curve, log ROC curve or LogAUC of one set over the others. The most common method uses ligands over property-matched decoys. If the target performs well, the general attitude is that the prospective virtual screen will perform well [22].

The ligands vs. decoys enrichment calculation was chosen as a codename for a way of evaluating how well the docking program performed against a target of interest (or a set of targets). Decoys refer to a set of molecules that (probably) will not bind to the target. Still, they are selected in such a way as to match the physico-chemical properties of the reference molecules closely (e.g., MW, LogP, HBA, HBD, etc.). Ultimately, if the ligands’ docking score outperforms the docking score of the decoys, this fact is a good indication that the model has a certain prediction power, allowing one to discriminate between them.

### 2.3. Molecular Docking, Verification of Docking Models

We performed molecular docking with low and standard docking effort levels and the above-discussed grids (Section 2.2), namely:

1. Sphere near TYR510—any hydrophobic atom (20 patterns);

2. H-bond Zn^2+^—with any hydrogen-acceptor atom (30 patterns);

3. ARG273—The first model of the “grid” (Grid-1), with any atom-acceptor of the hydrogen bond. The second model of the “grid” (Grid-2) has a different oxygen atom in both spheres.

Four sets of parameters were submitted for simulation:

Grid-1 and docking effort level 1.

Grid-2 and docking effort level 1.

Grid-1 and docking effort level 5.

Grid-2 and docking effort level 5.

All of these were further evaluated by calculating an enrichment factor. Accumulation curves are widely used to display ranking performances. Many metrics are currently employed to evaluate the performance of ranking methods in virtual screening (VS), for instance, the area under the receiver operating characteristic curve (ROC) and the area under the accumulation curve (AUAC). The AUAC can be interpreted as the probability that an active compounds defined by the rank-ordered list will be ranked before a compound randomly selected from a uniform distribution. The value is bounded between 1 and 0, with 1 being the ideal screen performance [23].

**Figure 5:**
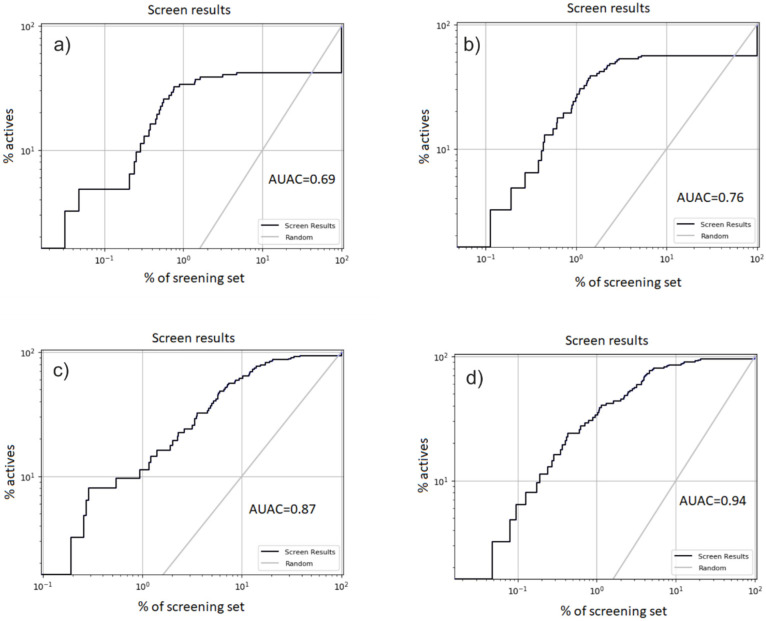
ROC curves of four docking results. (**a**) The first set: Grid-1—docking effort level 1. Area under accumulation curve: 0.69. (**b**) The second set: Grid-2—docking effort level 1. Area under accumulation curve: 0.76. (**c**) Grid-1—docking effort level 5. Area under accumulation curve: 0.87. (**d**) Grid-2—docking effort level 5. Area under accumulation curve: 0.94.

Next, we performed the enrichment evaluation of the screening, which was carried out with a set of active ligands and a set of decoys. The calculated area under the accumulation curve (AUAC = 0.94) indicated that we built near-optimal models. Comparing the results, we can see that the spherical positional constraints near ARG273 lead to a more accurate model for docking compared to the ARG273-H-bond for both docking efforts. Therefore, taking this into account, we chose Grid-2 to be the best model for further large-scale screening runs (Figure 5).

### 2.4. The Screening Workflow for the “Enamine Stock Compound Collection” (Enamine SCC, 3.2 Million Compounds)

The docking of ultra-large libraries opens up new opportunities and simultaneously brings new challenges. Docking tests the fit of each library molecule in a protein binding site in a process that often involves the sampling of hundreds-of-thousands to millions of possible configurations. To be feasible for a billion-molecule library on moderately sized computer clusters (e.g., 500–1000 cores), this calculation must consume not much more than 1 s/molecule/core. This need for speed means that the calculation cannot afford the level of detail and the number of interaction terms that would be necessary to achieve chemical accuracy [24]. This part of the work aims to develop and test the workflow for a medium-sized library for resource intensity, to further elaborate an effective approach to large-scale docking, in our case for the screening of the Enamine REAL database set (1.4 bln).

**Figure 6:**
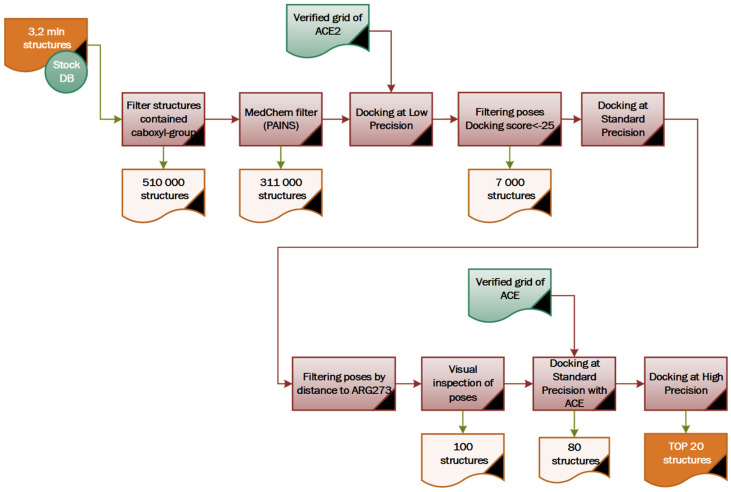
Virtual screening workflow for Enamine SCC (3.2 million compounds).

When dealing with enormous datasets, which is a part of this virtual screening study, optimizing available computational resources while retaining prediction accuracy becomes a decisive factor in building a project workflow. In this study, we modified the general approach recently proposed by Halgren and co-workers for large database screening [25]. Therefore, in order to screen Enamine SCC, we used the following stepwise algorithm (Figure 6):

- Filtering by the SMARTS substructure, looking for molecules with at least one carboxylic group;
- Applying a set of standard med-chem filters: PAINS (fragments that are listed as PAINS), BRENK (potential toxic reasons), NIH and ZINC (unwanted functional groups) and LILLY (potentially reactive or promiscuous compounds) [26,27];
- Performing molecular docking at a minimal level of theory with tracking of the conditional assessment of the interaction for critical amino acid residues;
- Molecular docking at medium accuracy and then ranking results according to the interaction energy of the ligands with the key residues of the target;
- Conducting a visual inspection of the poses and selecting a set of 100 molecules with the best positioning in the binding pocket.

**Figure 7:**
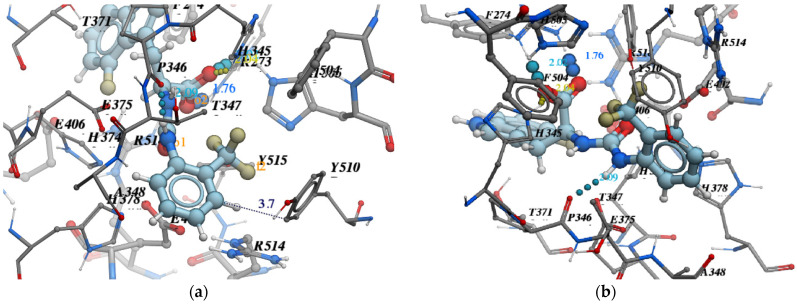
Important selection criteria for “cherry-picking”: (**a**) the position of the ligand’s hydrophobic moiety near TYR510; (**b**) the presence of hydrogen bond with PRO346.

**Figure 8:**
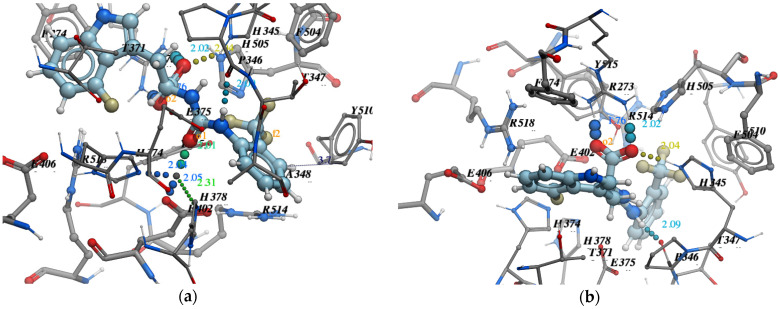
Important selection criteria for “cherry-picking”: (**a**) pose accuracy relative to ARG273; (**b**) pose accuracy relative to Zn^2+^.

The essential points which were taken into account during “cherry-picking” were:

- Pose accuracy and relative distance to ARG273;
- Pose accuracy and relative distance to Zn^2+^;
- The position of the ligand’s hydrophobic moiety in close proximity to TYR510 (Figure 7 and Figure 8); and
- The presence of a hydrogen bond with PRO346.

In the virtual screening workflow with ESCC (3.2 million compounds), the top 100 compounds were ultimately selected for docking against ACE. The selectivity evaluation accompanied by one more round of docking at the high precision level, followed by a visual inspection, allowed us to narrow down the selection to 20 candidate compounds (see Supplementary Materials, Table S1).

**Figure 9:**
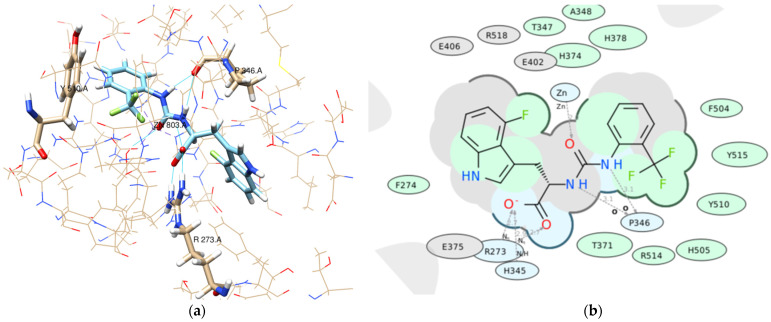
(**a**) 2d diagram of the interaction of Z4221819990 and ACE2 in the active site; (**b**) 3d visualization of the interaction of Z4221819990 and ACE2 in the active site.

For example, we considered the pose of the ligand **Z4221819990** (score −40.6) (Figure 9).

- Pose accuracy and relative distance to ARG273—the hydroxyl group binds the guanidine group ARG273 like a claw, which results in a perfect H-bond;
- Pose accuracy and relative distance to Zn^2+^—the oxygen atom binds the zinc ion, resulting in metal coordination between the partial negative charge on the oxygen atom and positively charged Zn^2+^;
- The position of the ligand’s hydrophobic moiety in close proximity to TYR510; the hydrophobic pocket near tyrosine (TYR510) is filled with an ortho-trifluorophenyl substitute;
- The presence of a hydrogen bond with PRO346—the hydrogen bond between proline and nitrogen is evident.

### 2.5. Screening the Enamine REAL Database Set (Enamine RDB, 1.4 Billion Compounds)

Virtual screening (VS) methods are the current trend in medical research for de novo drug discovery [28]. Large-scale VS is nowadays a standard step before wet-lab experiments in drug discovery [29,30]. Large-scale computing technologies have enabled high-throughput virtual screening involving thousands to billions of drug candidates. However, it is not trivial for biochemical scientists to evaluate technical alternatives and their implications for the running of such large experiments [31]. It is crucial to use the time of computational teams and the available computational resources optimally. When we plan a few smart workflows and streamline screening processes, one can empower computational chemists and our computational facilities to work smarter, not harder. Virtual screening management involves the organization, administration and governance of large volumes of both structured and unstructured data. Its goal is to ensure a high level of screening results using a reasonable amount of resources.

Our efforts in virtual screening management using experimental data were based on the following. The overall CPU time spent on the screening of a ligand library can be improved through the optimization of the screening plan by combining different methods and screening tools. The idea behind it this to find a delicate balance between accurate but computationally expensive methods and rough but computationally cheap ones.

To benchmark our docking calculations and estimate the potential for scaling, we used a small set of test molecules. Therefore, we docked a set of 250 compounds (prepared in 3D-sdf format) using only one CPU core at three different levels of effort: low-level **1**, standard-level **5** and high-level **10**. The benchmark results are shown in Table 1.

Furthermore, we planned and assessed the CPU time needed to implement each of the three screening plans. Detailed descriptions of roadmaps RM1, RM5 and RM10 are provided in Table 2, Table 3 and Table 4.

The benefits of proper scheduling of a screening pipeline are compared in Table 5.

As can be seen from the calculations, the correct screening roadmap and smart management can dramatically save CPU time (up to 2000 times). Therefore, we chose RM3, which showed the best resource costs and efficiency results. Using the screening protocol, RM3 will allow us to screen even more extensive libraries of a size up to 40 billion compounds. For example, on an ordinary compute node of 40 cores, Enamine RDB, with 1.4 billion compounds, was processed in 25 h. Thus, the approximate time required for processing a library of 40 billion compounds would be 721 h (about a month). This timeframe is in total agreement with the regular practice of large-scale screening campaigns in drug discovery. At the same time, following the RM1 screening protocol on a 40-core node would take about 662,024 h (86 months), and following the RM2 protocol it would take 6213 h (8 months), respectively. It should be noted that the above calculations are estimates and do not consider human factors and technical failure risks.

**Figure 10:**
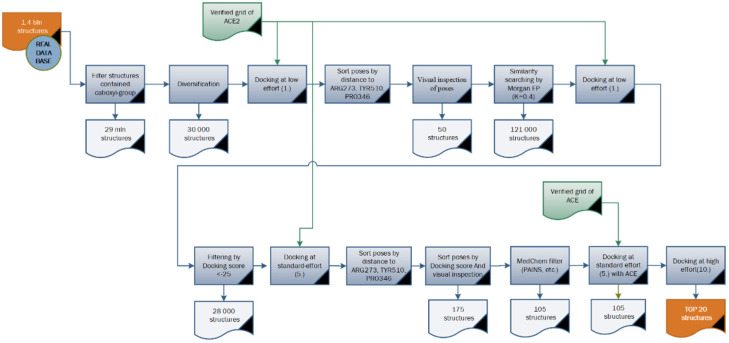
Virtual screening workflow for Enamine RDB (1.4 billion compounds).

Screening of the Enamine RDB was performed using an algorithm chosen from the upper estimation. It was performed using a similar algorithm with minor modifications (Figure 10):

- After the first SMARTS filtering (for the existence of carboxylic groups), 29 million compounds were found. Considering the size of the set, we used the chemical diversity approach (Tanimoto similarity between the molecules represented via Morgan fingerprints [32]). This narrowed down the set to 30,000 substances. After performing the docking of the 30K-set, 50 compounds were selected based on their docking score and visual inspection results. For these compounds, we performed a shape-based similarity search by means of the USRCAT (Ultrafast Shape Recognition with Credo Atom Types) method [33] and then selected those with a Tanimoto similarity of 0.4 or above. Our screening set at this step became larger, with 121K compounds.
- The next step was molecular docking at the low-effort level **1** using a verified model (Section 2). The results were filtered by a docking score < −25, leaving 28,000 ligands.
- Molecular docking at the standard-effort level **5** using a verified model was the next step in our screening. For a more productive virtual view, it was also first filtered by the value of closest distance in the contact analyzer (ARG273), (TYR510), (PRO346). Then the library was sorted by docking score and “cherry-picking” using the same value criteria as for working with the stock base.
- After the calculation of ChemFilters (PAINS, LILLY, IN, USA) and the screening of compounds, we were left with 105 ligands.
- To verify the possible non-selectivity of ligands in relation to ACE, we carried out molecular coupling of the obtained TOP105 compounds with the ACE target. Only one compound showed a score of more than 25, but the pose in it was unnatural. Therefore, we did not discard these compounds.
- 105 compounds were investigated via molecular docking at a high level of effort (level **10**) with high accuracy. The model used was the same as the one used previously in this work. Through an expert evaluation, paying attention to the score values, the 20 top compounds were found for specific interactions with important residues.

Finally, the top 105 compounds selected from the RDB set were treated as described above, resulting in 20 promising hits (see Supplementary Materials, Table S2).

### 2.6. Molecular Dynamics Study

Finally, molecular dynamics simulations of selected ligands with a duration of 10 ns showed the good stability of the host-guest complexes, which indicated significant chances of demonstrating in vitro activity. The molecular dynamics modelling scheme considered the available computing power. The simulation size (number of particles), time step and total time duration were chosen so that the calculation could be completed within a reasonable period. However, the simulation was long enough to match the timescale of the natural processes being studied. To draw statistically valid conclusions from the simulation, the time of the simulated period corresponded to the kinetics of the natural process.

That is why we decided to carry out molecular dynamics calculations with a duration of 10 ns. The aim was a more reliable justification of the selected ligands and the evidence that important interactions are maintained for an extended period during the evolution of the system. The top three ligands were selected for molecular dynamics studies.

During the analysis of the MD trajectory, our primary focus was on the following:

(1) The RMSD of the ligand position in the complex during the MD run;
(2) The estimation of the number of key contacts between the ligand and protein at each point during the dynamic period; and
(3) Lifetime evolution of specific ligand–protein interactions along the MD trajectory.

**Figure 11:**
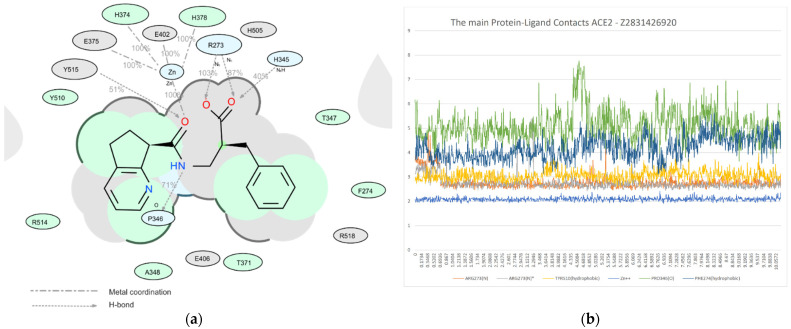
A detailed interaction scheme of ligand atoms (Z2831426920) with protein residues: (**a**) generalized graphical representation of the interactions and contacts (H-bonds, hydrophobic, ionic, water bridges); (**b**) scheme showing the distance evolution (in Å) between the ligand and residues.

**Figure 12:**
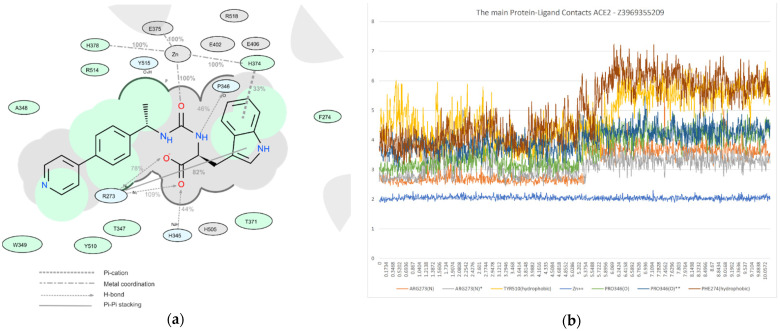
A detailed interaction scheme of ligand atoms (Z3969355209) with protein residues: (**a**) generalized graphical representation of the interactions and contacts (H-bonds, hydrophobic, ionic, water bridges); (**b**) scheme showing the distance evolution (in Å) between the ligand and residues.

**Figure 13:**
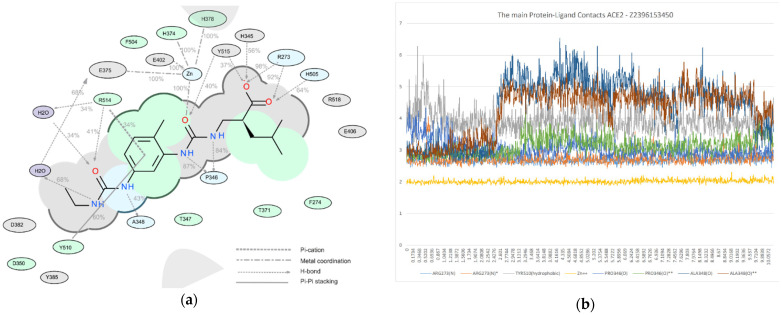
A detailed interaction scheme of ligand atoms (Z2396153450) with protein residues: (**a**) generalized graphical representation of the interactions and contacts (H-bonds, hydrophobic, ionic, water bridges); (**b**) scheme showing the distance evolution (in Å) between the ligand and residues.

Among all protein–ligand interactions (or “contacts”), we focused on the following four types: hydrogen bonds, metal coordination, Pi–cation and Pi–Pi stacking. Composite bar charts are normalized over a trajectory; for example, a value of 0.7 means that a specific interaction is present during 70% of the simulation time. Values greater than 1.0 are possible because some protein residues may form multiple contacts of the same subtype with the ligand. We have presented schemes of the detailed interactions of ligand atoms with protein residues in Figure 11, Figure 12 and Figure 13. Interactions were found to occur during more than 30.0% of the simulation time in the selected trajectory (from 0.0 to 10.2 ns).

**Figure 14:**
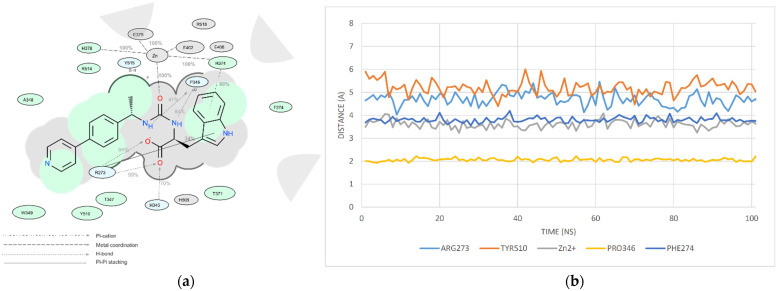
A detailed interaction scheme of ligand atoms (Z3969355209) with protein residues (100 ns): (**a**) generalized graphical representation of the interactions and contacts (H-bonds, hydrophobic, ionic, water bridges); (**b**) scheme showing the distance evolution (in Å) between the ligand and residues.

We decided to confirm the validity of our choice for the duration of the molecular dynamics simulations of the selected ligands (10 ns). For this purpose, we performed similar research with the duration of 100 ns using one of the top ligands (Z3969355209). The results are summarized in Figure 14.

**Figure 15:**
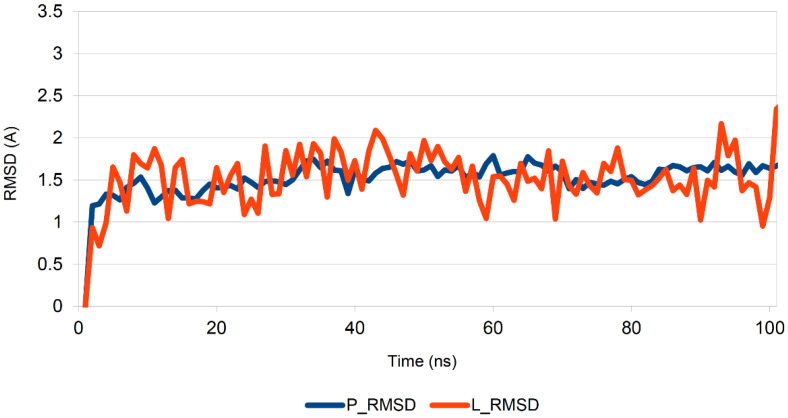
Protein and ligand RMSD plots of the complex (ACE2-Z3969355209).

Furthermore, the root-mean-square deviation (RMSD) of certain atoms in a molecule with respect to a reference structure was calculated via least-square fitting of the structure to the reference structure. The blue graph (P_RMSD) shows the evolution of the proteins’ RMSD. All protein frameworks in the first place were aligned to the frame backbone and then the RMSD was computed. Changes on the order of 1–3 Å are completely acceptable for small globular proteins. The ligand RMSD (L_RMSD) indicates how stable the ligand is, concerning the protein and its binder pocket. If the observed values are significantly greater than the RMSD of the protein, then it is likely that the ligand diffused from its original binding site. In our case, the protein and ligand RMSD values did not exceed 2.5–3 A from the initial frame, which indicates the stability of the complex (Figure 15) [34].

**Figure 16:**
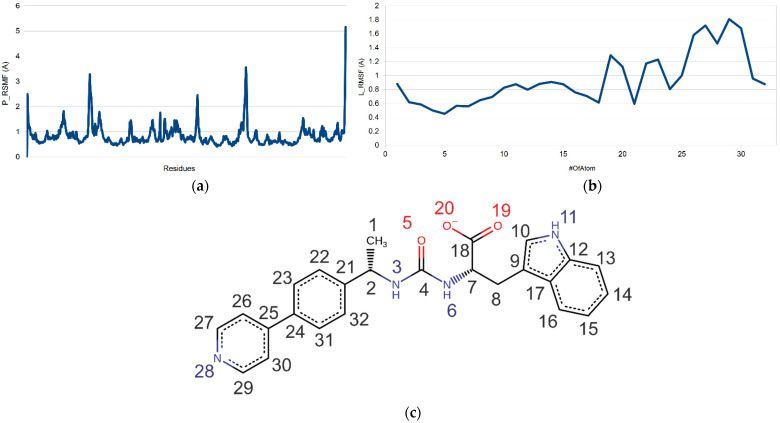
RSMF plots of (**a**) protein, (**b**) ligand Z3969355209 and (**c**) respective atomic enumeration in the ligand.

Furthermore, we calculated root-mean-square fluctuation (RMSF) as a useful indicator of local changes in the ligand atom positions and along the protein chain. The standard deviation (RMSF) was constructed. The peaks in this plot indicate areas of the protein that fluctuated the most during the simulation [35]. The RMSF ligand explains how the ligand fragments interact with the protein and their entropic role in the binding event. Figure 16 shows these changes in proteins (a) and ligands (b) according to atomic enumeration in the ligand.

## 3. Materials and Methods

### 3.1. Molecular System Setup for Docking Simulation

#### 3.1.1. Protein Preparation

In this work, we used the following X-ray structures of the targets ACE2 (PDB code: 1R4L) and ACE (PDB code: 1O86) [13].

Firstly, we converted the proteins as chemicals into ICM objects, then deleted all water molecules. Empirically appended hydrogen atoms were further optimized, together with the protein structures, through a built-in structure optimization module. Furthermore, we optimized the orientation of HIS, PRO, ASN, GLN and CYS. The following residues were further optimized: HIS—three protonation states and two rotations were checked and the residue was renamed according to its subtype, HIE (epsilon tautomer) or HIP (+). ASN and GLN—(a 180-degree flip was tried).

#### 3.1.2. Ligand Preparation

All reference structures were selected to test docking models from the ChEMBL database (https://www.ebi.ac.uk/chembl/, accessed on 21 July 2021).

Commercially available collections (Enamine SCC, 3.2 million compounds) and an “on-demand” database (Enamine RDB, 1.4 billion compounds) were used.

The ligand preparation procedure was performed by including hydrogens, followed by the minimization of ligand structures (performing 3D structure rendering if necessary). Then we generated all the ligand-protonated states found in the specified pH range 5.0–9.0. The next steps in ligand preparation involved the generation of the lowest-energy ring conformations only, desalting and the generation of tautomers and stereoisomers (two stereoisomers per chiral center in the ligand).

#### 3.1.3. Molecular Docking

In silico screening was performed using the Molsoft 3.8.6 program package [36], accompanied by the Chimera1.14 visualization program [37].

### 3.2. Molecular Dynamics Simulation

Classical molecular dynamics (MD) simulations allow the implementation of SBDD strategies that fully account for the structural flexibility of the overall drug−target model system [38].

Indeed, it is now widely accepted that the two major drug-binding paradigms (induced-fit and conformational selection) have superseded Emil Fischer’s rigid lock-and-key binding paradigm. Receptor and ligand flexibility are crucial for correctly predicting drug binding and related thermodynamic and kinetic properties [37].

The molecular dynamics were calculated using Gromacs 2019 software (Version 2019.5). Solvent model—SPC, box shape—orthorhombic, size—10 Å × 10 Å × 10 Å, Force fieldcharmff3631. Neutralization was accomplished by adding Na^+^ ions. Simulation time—10 ns. Number of frames—1000. Ensemble class—NPT. Temperature—300 K. Pressure (bar)—1.01325. Thermostat method—Nosé–Hoover chain. We relaxed the model system before simulation, with a relaxation time of 1 ps. Interaction cutoff radius—9 Å.

## 4. Conclusions

We modelled the ACE2 and ACE receptors and the models showed great prediction power against a set of publicly available binders with known experimental activity. These results allowed us to evaluate ACE2/ACE selectivity in silico, which is a crucial checkpoint for developing chemical probes for ACE2. Furthermore, we built chemical diversity-applied workflows that enabled us to deal with huge compound collections, which was demonstrated on the docking of 1.4 billion compounds cost-effectively. The chemo-type-based approach allows us to fish out small sets of molecular candidates with good potential for further in vitro optimization.

As demonstrated by our MD studies, all compounds showed stable ligand–protein interactions along their MD production trajectories. Therefore, we found these compounds to have a great potential to be active during in vitro testing.

In this work, we have also reviewed possible optimization protocols for virtual screening management and used experimental data to illustrate the following point: the overall CPU time spent on the screening of a ligand library can be improved through optimization of the screening plan as a combination of different methods and screening tools, including both accurate and more superficial tools. As we can see from the calculations, a proper screening roadmap and smart management can reduce the CPU time by up to 10 times.

## Figures and Tables

**Table 1:** Measurement results of computing capabilities ^1^.

Level of Effort	CPU	Number of Ligands	Time, min	Min per Ligand
1	1	250	31	0.124
5	1	250	110	0.44
10	1	250	220	0.88

^1^ Technical information for the testing workstation on which the measurements were made: 1. CPU: Intel (R) Core (TM) i5-9600K CPU @ 3.70 GHz; 2. 16 GB of RAM; 3. Operating System: Ubuntu 20.04.3 LTS.

**Table 2:** Basic roadmap RM1.

Description of the Step	Number of Structures Left	CPU Time (min)
INPUT	1.41 bln	-
MedChem filter (PAINS, etc.)	1.2 bln	1800
Ligand docking at low effort (1.)	100,000	148,800,000
Ligand docking at standard effort (5.)	10,000	44,000
Ligand docking at standard effort (5.) with ACE	9000	4400
Ligand docking at high effort (10.)	200	7920
Sort pose by docking score and visual inspection	20	-
Total CPU time (min)	-	148,858,120

**Table 3:** Advanced roadmap RM2.

Description of the Step	Number of Structures Left	CPU Time (min)
INPUT	1.41 bln	-
Diversification 1/10	141 mln	1800
MedChem filter (PAINS, etc.)	120 mln	1800
Ligand docking at low effort (1.)	-	14,880,000
Sort poses by distance to ARG273, TYR510, PRO346	50,000	-
Ligand docking at standard effort (5.)	-	22,000
Sort poses by distance to ARG273, TYR510, PRO346	5000	-
Ligand docking at standard effort (5.) with ACE	4500	2200
Ligand docking at high effort (10.)	100	3960
Sort poses by docking score and visual inspection	20	-
Total CPU time (min)	-	14,909,960

**Table 4:** Optimal roadmap RM3.

Description of the Step	Number of Structures Left	CPU Time (min)
INPUT	1.41 bln	-
Filter structures contained carboxyl-group	141 mln	600
Diversification	120 mln	600
Ligand docking at low effort (1.)	-	3720
Sort poses by distance to ARG273, TYR510, PRO346	50,000	-
Visual inspection of poses	-	-
Similarity searching by Morgan FP	5000	2250
Ligand docking at standard effort (5.)	4500	53,240
Sort poses by distance to ARG273, TYR510, PRO346	100	
Sort poses by docking score and visual inspection	20	-
MedChem filter (PAINS, etc.)		-
Ligand docking at standard effort (5.) with ACE		46.2
Ligand docking at the level of effort (10.)		92.4
Total CPU time (hours)	-	60,548.6

**Table 5:** Benefits of proper scheduling of a screening pipeline.

Comparison of the Estimations of CPU Time Required for Roadmaps	Difference (Number of Times)
RM1 vs. RM2	10
RM1 vs. RM3	2458
RM2 to RM3	246

## Data Availability

The data presented in this study are available on request from the corresponding author.

## Supplementary Materials

The following are available online. List S1: List of ligands from the ChEMBL database with experimentally measured activities—Ki or IC50—against ACE2; Table S1: Top 20 compounds from Enamine SSC, showcasing visualizations of poses in the ACE2 binding pocket; Table S2: Top 20 compounds from Enamine RDB, showcasing visualizations of poses in the ACE2 binding pocket.

## Sample Availability

Samples of the compounds are not available from the authors.
