# Supplementary material for: Virtual Screening in Search for a Chemical Probe for Angiotensin-Converting Enzyme 2 (ACE2)
Source: Molecules. 2021 Dec 14;26(24):7584. doi: 10.3390/molecules26247584 (PMC8707431; doi:10.3390/molecules26247584)
Supplement: Supplementary file 1 [file molecules-26-07584-s001.zip › molecules-1476357-supplementary.pdf]

Virtual screening in search for a chemical probe for angiotensin-converting enzyme 2 (ACE2)

Supporting Information

List S1. List if ligands from the ChEMBL database with experimentally measured activities Ki or IC50 against ACE2

Smiles Molecule ChEMBL ID

CC[C@@H](C)[C@H](S)C(=O)N[C@@H](Cc1ccc(O)cc1)C(=O)O CHEMBL253224

CC[C@@H](C)[C@H](S)C(=O)N[C@@H](Cc1cccc(Oc2ccccc2)c1)C(=O)O CHEMBL252003

CCCC[C@H](S)C(=O)N[C@@H](Cc1ccc(-c2ccccc2)cc1)C(=O)O CHEMBL257270

CC[C@H](S)C(=O)N[C@@H](Cc1ccc(-c2ccccc2)cc1)C(=O)O CHEMBL257726

O=C(CS)N[C@@H](Cc1ccc(-c2ccccc2)cc1)C(=O)OCHEMBL258333

O=C(O)[C@H](Cc1ccc(-c2ccccc2)cc1)NC(=O)[C@H](S)Cc1ccccc1 CHEMBL163454

CC[C@@H](C)[C@H](S)C(=O)N[C@@H](Cc1ccccc1OCc1ccccc1)C(=O)O CHEMBL254493

CC[C@@H](C)[C@H](S)C(=O)N[C@@H](Cc1cccc2ccccc12)C(=O)O CHEMBL400527

CC[C@@H](C)[C@H](S)C(=O)N[C@@H](Cc1ccc(OCc2ccc(F)cc2F)cc1)C(=O)O CHEMBL398771

CC[C@@H](C)[C@H](S)C(=O)N[C@@H](Cc1ccc(OCc2ccc(F)cc2)cc1)C(=O)O CHEMBL254911

CC[C@@H](C)[C@H](S)C(=O)N[C@@H](C)C(=O)O CHEMBL254900

CC[C@@H](C)[C@H](S)C(=O)N[C@@H](Cc1ccc(-c2ccccc2)cc1)C(=O)O CHEMBL252391

CC[C@@H](C)[C@H](S)C(=O)N[C@@H](Cc1ccc(OCc2cc(C(F)(F)F)cc(C(F)(F)F)c2)cc1)C(=O)O CHEMBL252009

CC[C@H](C)[C@H](S)C(=O)N[C@@H](Cc1ccc(-c2ccccc2)cc1)C(=O)O CHEMBL269996

CC[C@@H](C)[C@H](S)C(=O)N[C@@H](Cc1cccc(-c2ccccc2)c1)C(=O)O CHEMBL401397

CC[C@@H](C)[C@H](S)C(=O)N[C@@H](Cc1ccccc1)C(=O)O CHEMBL400526

CC[C@@H](C)[C@H](S)C(=O)N[C@@H](Cc1cccc(OCc2ccccc2)c1)C(=O)O CHEMBL254495

CC(C)C[C@H](S)C(=O)N[C@@H](Cc1ccc(-c2ccccc2)cc1)C(=O)O CHEMBL271225

CC[C@@H](C)[C@H](S)C(=O)N[C@@H](Cc1ccccc1-c1ccccc1)C(=O)O CHEMBL398545

CC[C@@H](C)[C@H](S)C(=O)N[C@@H](Cc1ccc(Oc2ccccc2)cc1)C(=O)O CHEMBL254282

O=C(O)[C@H](Cc1ccc(-c2ccccc2)cc1)NC(=O)[C@@H](S)C1CCCC1 CHEMBL404117

O=C(N[C@@H](Cc1ccc(-c2ccccc2)cc1)C(=O)O)[C@@H](S)Cc1ccccc1 CHEMBL350414

O=C(N[C@@H](Cc1ccc(-c2ccccc2)cc1)C(=O)O)[C@@H](S)CC1CCCCC1 CHEMBL271224

CC(C)(C)C[C@H](S)C(=O)N[C@@H](Cc1ccc(-c2ccccc2)cc1)C(=O)O CHEMBL271223

O=C(O)[C@H](Cc1ccc(-c2ccccc2)cc1)NC(=O)[C@@H](S)C1CCCCC1 CHEMBL257026

CC(=O)N[C@@H](Cc1c[nH]cn1)C(=O)N[C@H](CC(C)C)P(=O)(O)CC(Cc1cc(-c2ccccc2)no1)C(=O)O CHEMBL408182

CC(C)C[C@H](NC(=O)[C@@H]1CCCN1C(=O)[C@H](CO)NC(=O)[C@H](CS)NC(=O)[C@H](Cc1cnc[nH]1)NC(=O)[C@H](CO)NC(=O)[C@H](Cc1ccc(O)cc1)NC(=O)[C@@H](CC(=O)O)NC(=O)CN)C(=O)N[C@@H](CCCNC(=N)N)C(=O)N[C@@H](Cc1ccc(O)cc1)C(=O)N[C@@H](Cc1ccc(O)cc1)C(=O)N1CCC[C@H]1C(=O)N[C@@H](Cc1c[nH]c2ccccc12)C(=O)N[C@@H](Cc1c[nH]c2ccccc12)C(=O)N[C@@H](CCCCN)C(=O)N[C@@H](CS)C(=O)N[C@H](C(=O)N[C@@H](Cc1ccc(O)cc1)C(=O)N1CCC[C@H]1C(=O)N[C@@H](CC(=O)O)C(=O)N1CCC[C@H]1C(=O)O)[C@@H](C)O CHEMBL1240682

CC(=O)N[C@@H](CC(C)C)C(=O)N1CCC[C@@H]1P(=O)(O)CC(Cc1cc(-c2ccccc2)no1)C(=O)O CHEMBL261121

CC(C)C[C@@H](N)P(=O)(O)C[C@@H](Cc1ccccc1)C(=O)O CHEMBL405232

CC(C)(S)C(=O)N[C@@H](Cc1ccc(-c2ccccc2)cc1)C(=O)O CHEMBL404044

C[C@H](S)C(=O)N[C@@H](Cc1ccc(-c2ccccc2)cc1)C(=O)OCHEMBL257727

CC[C@@H](C)[C@H](S)C(=O)N[C@@H](Cc1ccc(OCc2ccc(C(F)(F)F)cc2)cc1)C(=O)O CHEMBL251809

CC[C@@H](C)[C@H](S)C(=O)N[C@@H](Cc1ccccc1Oc1ccccc1)C(=O)O CHEMBL251804

CC(C)C[C@@H](NC(=O)OCc1ccccc1)P(=O)(O)C[C@@H](Cc1ccccc1)C(=O)O CHEMBL264665

O=C(N[C@@H](Cc1ccc(-c2ccccc2)cc1)C(=O)O)[C@@H](S)Cc1ccc2ccccc2c1 CHEMBL272925

O=C(O)[C@H](Cc1ccc(-c2ccccc2)cc1)NC(=O)[C@@H](S)c1ccccc1 CHEMBL437595

O=C(O)[C@H](Cc1ccccc1)CP(=O)(O)[C@H]1CCCN1 CHEMBL261033

CC(=O)N[C@@H](Cc1c[nH]cn1)C(=O)N1CCC[C@@H]1P(=O)(O)C[C@@H](Cc1ccccc1)C(=O)O CHEMBL261423

CC(=O)N[C@@H](CCCCN)C(=O)N1CCC[C@@H]1P(=O)(O)C[C@@H](Cc1ccccc1)C(=O)O CHEMBL411942

C[C@@H](N)P(=O)(O)C[C@@H](Cc1ccccc1)C(=O)O CHEMBL405913

N[C@H](Cc1ccccc1)P(=O)(O)C[C@@H](Cc1ccccc1)C(=O)O CHEMBL412123

CC(=O)N[C@@H](CC(C)C)C(=O)N1CCC[C@@H]1P(=O)(O)C[C@@H](Cc1ccccc1)C(=O)O CHEMBL260273

C[C@H](CP(=O)(O)[C@H]1CCCN1C(=O)OCc1ccccc1)C(=O)O CHEMBL409713

CC(=O)N[C@@H](CCC(=O)O)C(=O)N1CCC[C@@H]1P(=O)(O)C[C@@H](Cc1ccccc1)C(=O)O CHEMBL263663

CC(=O)N[C@@H](Cc1c[nH]cn1)C(=O)N[C@H](CC(C)C)P(=O)(O)C[C@@H](Cc1ccccc1)C(=O)O CHEMBL408448

CC(=O)N[C@H](C(=O)N1CCC[C@@H]1P(=O)(O)C[C@@H](Cc1ccccc1)C(=O)O)C(C)C CHEMBL411052

CC(=O)N[C@@H](Cc1ccc(O)cc1)C(=O)N1CCC[C@@H]1P(=O)(O)C[C@@H](Cc1ccccc1)C(=O)O CHEMBL410509

CC(C)C[C@H](CP(=O)(O)[C@H]1CCCN1C(=O)OCc1ccccc1)C(=O)O CHEMBL411298

CC(=O)N[C@@H](Cc1c[nH]cn1)C(=O)N1CCC[C@@H]1P(=O)(O)CC(Cc1cc(-c2ccccc2)no1)C(=O)O CHEMBL409105

CC[C@@H](C)[C@H](S)C(=O)N[C@@H](Cc1ccc(OCc2ccccc2)cc1)C(=O)O CHEMBL254703

CC[C@@H](C)[C@H](S)C(=O)N[C@@H](Cc1ccc2ccccc2c1)C(=O)O CHEMBL253428

CC[C@@H](C)[C@H](S)C(=O)N[C@@H](Cc1ccc(OCc2ccc(F)c(F)c2)cc1)C(=O)O CHEMBL251808

CC[C@@H](C)[C@H](S)C(=O)NCC(=O)O CHEMBL401086

CC(=O)N[C@@H](CC(C)C)C(=O)N[C@H](Cc1ccccc1)P(=O)(O)C[C@@H](Cc1ccccc1)C(=O)OCHEMBL408705

CC(=O)N[C@@H](Cc1ccccc1)C(=O)N1CCC[C@@H]1P(=O)(O)C[C@@H](Cc1ccccc1)C(=O)O CHEMBL258698

O=C(N[C@H](Cc1ccccc1)P(=O)(O)C[C@@H](Cc1ccccc1)C(=O)O)OCc1ccccc1 CHEMBL258464

CC(=O)N[C@@H](C)C(=O)N1CCC[C@@H]1P(=O)(O)C[C@@H](Cc1ccccc1)C(=O)O CHEMBL409721

O=C(O)[C@@H](Cc1ccccc1)CP(=O)(O)[C@H]1CCCN1C(=O)OCc1ccccc1 CHEMBL260677

C[C@@H](NC(=O)OCc1ccccc1)P(=O)(O)C[C@@H](Cc1ccccc1)C(=O)O CHEMBL258683

O=C(N[C@@H](Cc1ccc(-c2ccccc2)cc1)C(=O)O)[C@@H](S)CCc1ccccc1 CHEMBL402987

O=C(O)[C@H](Cc1ccc(-c2ccccc2)cc1)NC(=O)[C@@H](S)C1CCC1 CHEMBL257229

CC(C)[C@H](S)C(=O)N[C@@H](Cc1ccc(-c2ccccc2)cc1)C(=O)O CHEMBL269997

CC(=O)CNC(=O)[C@H](CC(=O)O)NC(=O)[C@H](Cc1ccc(O)cc1)NC(=O)[C@H](CO)NC(=O)[C@H](Cc1c[nH]cn1)NC(=O)[C@@H]1CSSC[C@H](NC(=O)[C@@H](NC(=O)[C@H](Cc2ccc(O)cc2)NC(=O)[C@@H]2CCCN2C(=O)[C@H](CC(=O)O)NC(=O)[C@@H]2CCCN2C(=O)[C@H](CCC(=O)O)NC(=O)CNC(=O)CNC(=O)CN)[C@@H](C)O)C(=O)N[C@@H](CCCCN)C(=O)N[C@@H](Cc2c[nH]c3ccccc23)C(=O)N[C@@H](Cc2c[nH]c3ccccc23)C(=O)N2CCC[C@H]2C(=O)N[C@@H](Cc2ccc(O)cc2)C(=O)N[C@@H](Cc2ccc(O)cc2)C(=O)N[C@@H](CCCNC(=N)N)C(=O)N[C@@H](CC(C)C)C(=O)N2CCC[C@H]2C(=O)N[C@@H](CO)C(=O)N1 CHEMBL436639

Cc1nnc(S/C(=C/c2ccc(-c3cc(Cl)ccc3C)o2)C(=O)O)[nH]1 CHEMBL252417

CC(C)C[C@H](N[C@@H](Cc1cncn1Cc1cc(Cl)cc(Cl)c1)C(=O)O)C(=O)O CHEMBL429844

CNC(=O)[C@H](Cc1c[nH]cn1)NC(=O)CN(CCCc1ccccc1)CC(=O)O CHEMBL3235416

NCCNCCN1CC1 CHEMBL398940

Table S1. Top 20 compound from Enamine SSC, showcasing visualizations of poses in ACE2 binding pocket.

#	ID	Score	2d diagram of interaction	3d visualization of interaction
**1**	Z2831426920	-35,38	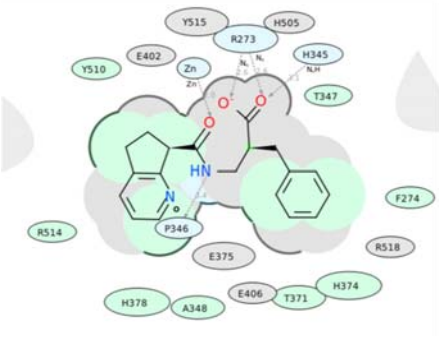	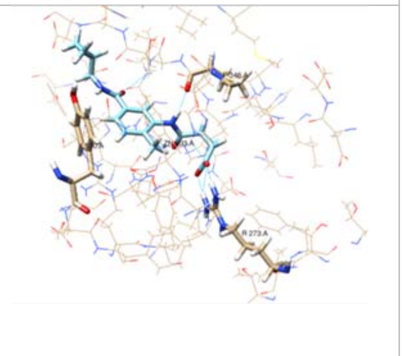

**2**	Z3681209542	-26,96	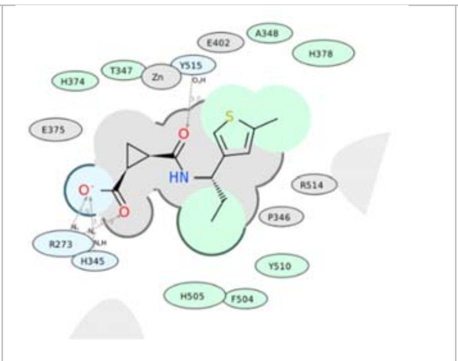	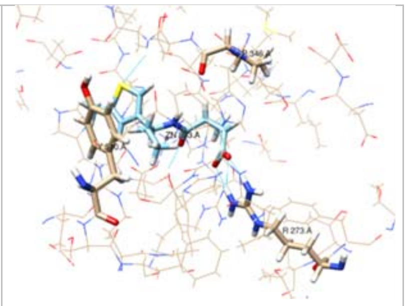
**3**	Z3681208436	-32,95	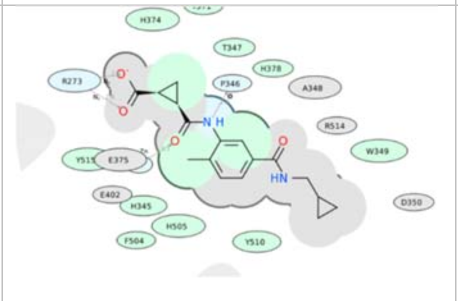	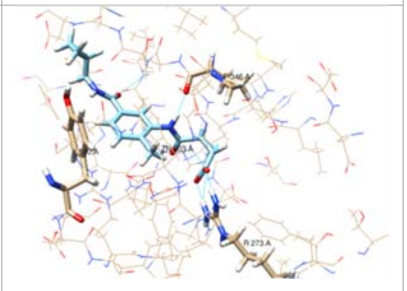
**4**	Z3681332013	-26,15	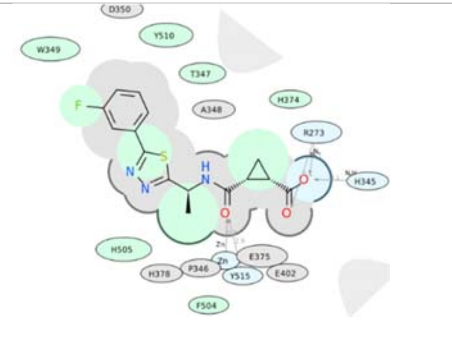	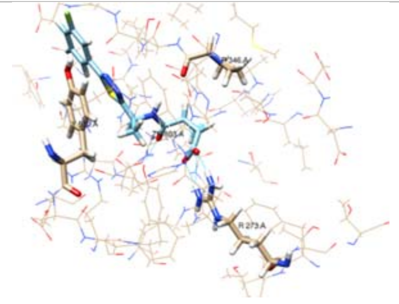
**5**	Z3969355209	-38,31	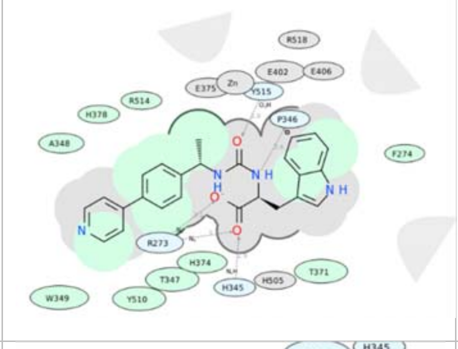	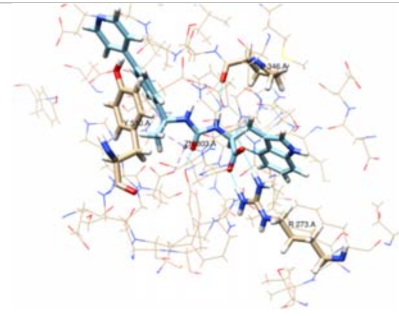
**6**	Z3681736600	-34,15	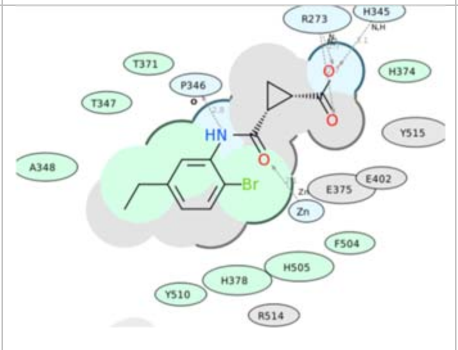	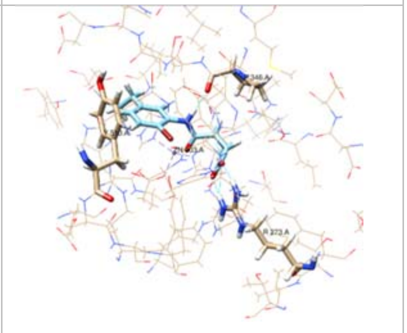

**7**	Z3681207877	-30,48	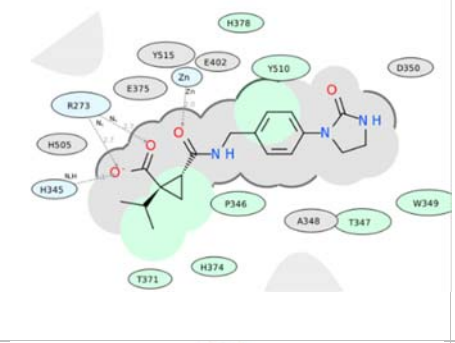	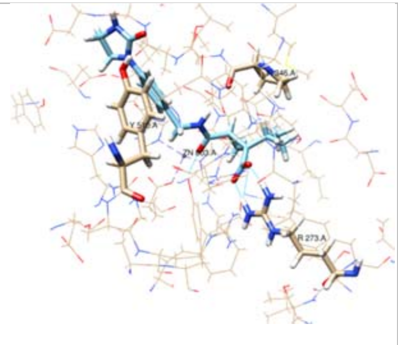
**8**	Z3681593370	-27,76	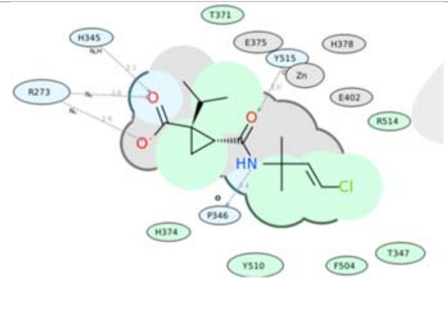	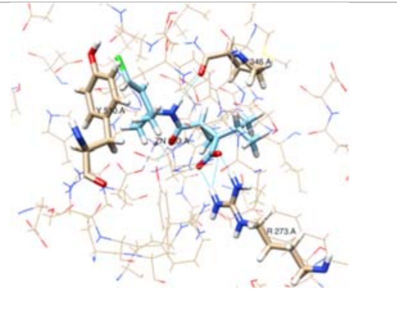
**9**	Z3681369109	-25,08	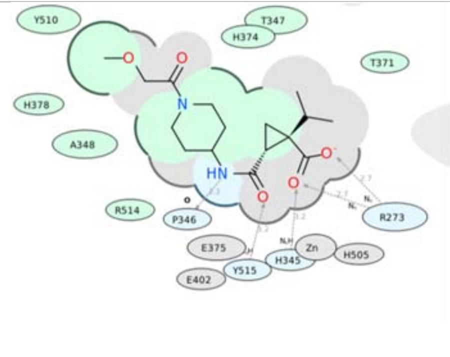	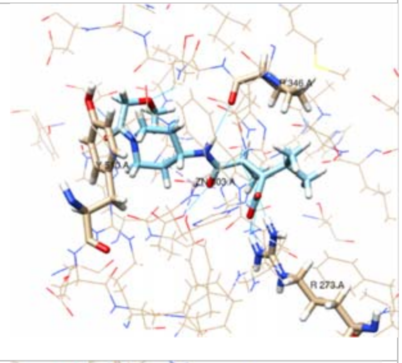
**10**	Z4188337437	-33,82	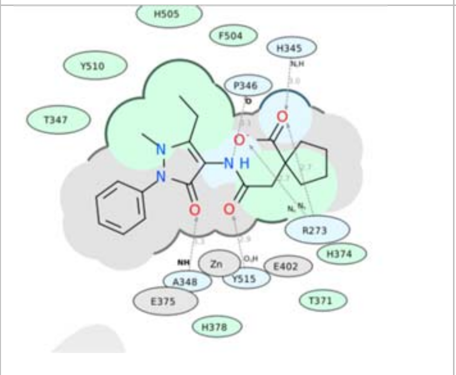	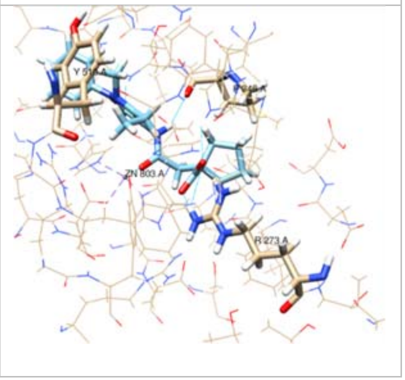

**11**	Z3037294184	-30,6	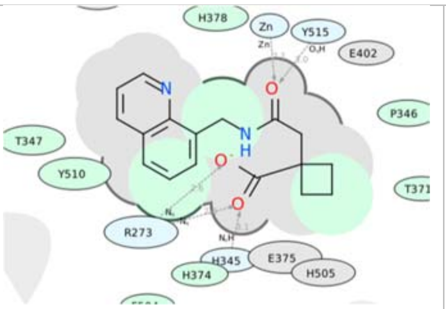	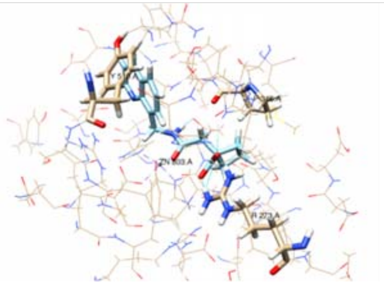
**12**	Z4549268235	-<br>34,46	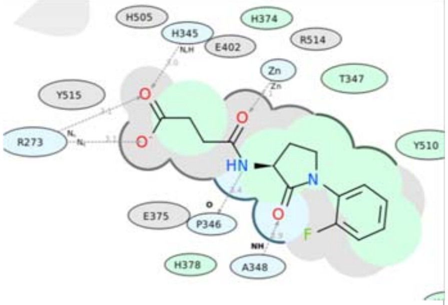	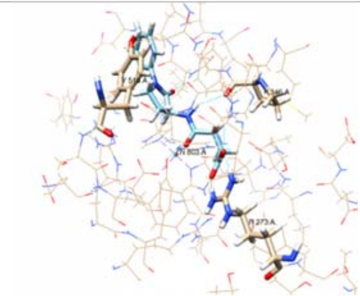
**13**	Z4361984504	-33.6	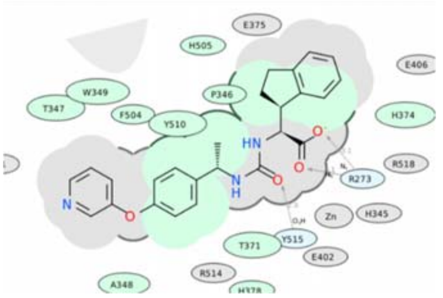	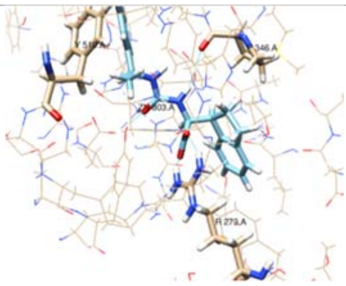
**14**	Z3681806363	-<br>35,03	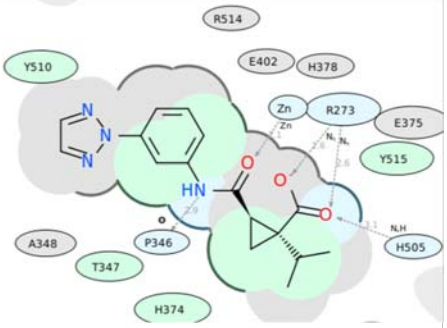	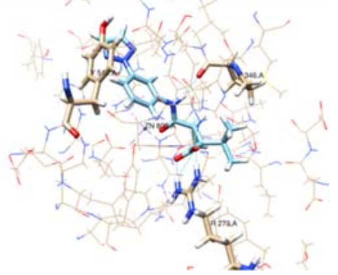
**15**	Z3681799936	-<br>22,59	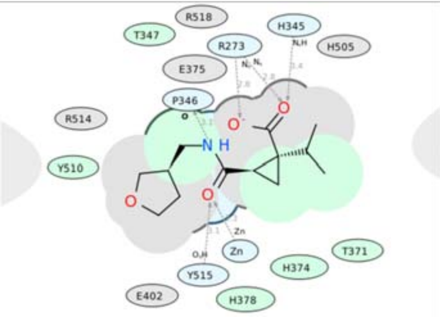	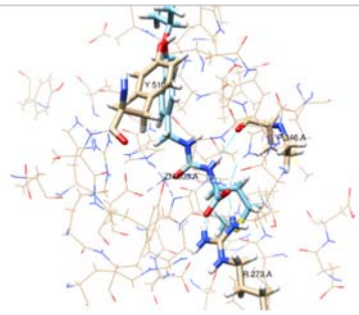

**16**	Z4602008618	-29	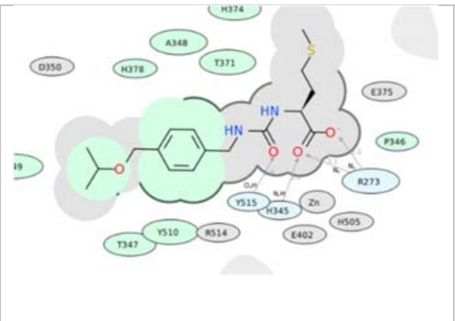	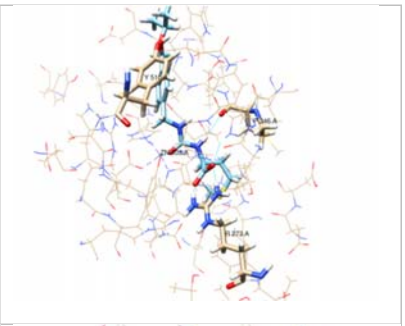
**17**	Z3466179106	-26,43	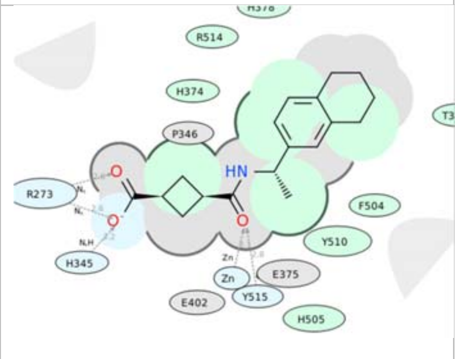	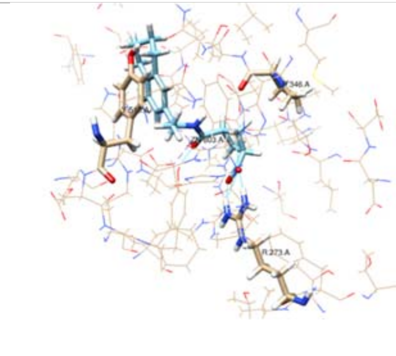
**18**	Z4221819990	-40,66	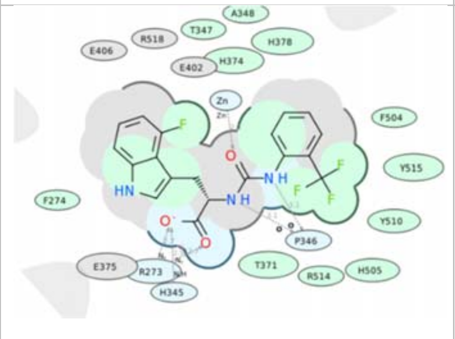	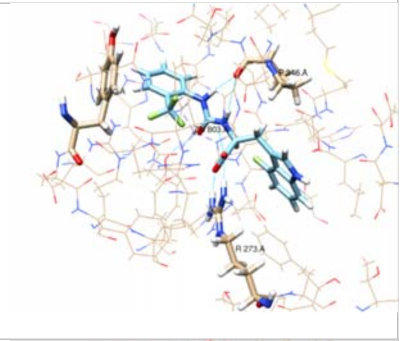
**19**	Z4561748167	-32,92	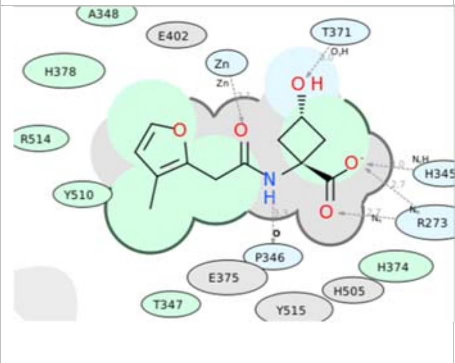	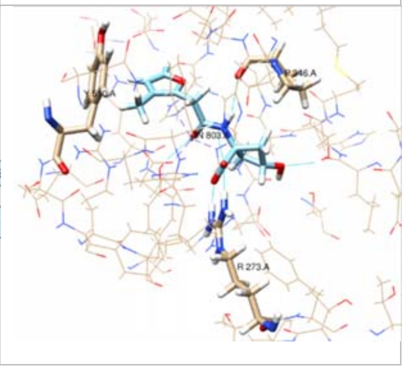

**20**	Z4119544425	-34,1	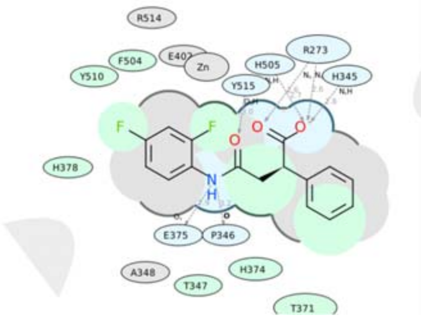	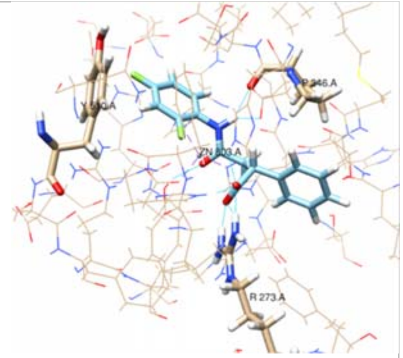

Table S2. Top 20 compounds from Enamine RDB, showcasing visualizations of poses in ACE2 binding pocket.

#	ID	Score	2d diagram of interaction	3d visualization of interaction
1	Z1991725061	-37.32	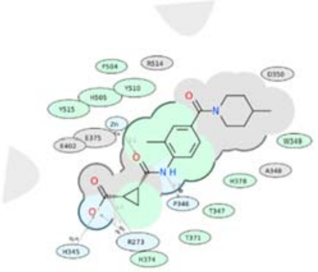	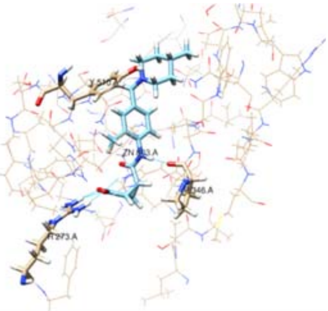
2	Z2396153450	-37.86	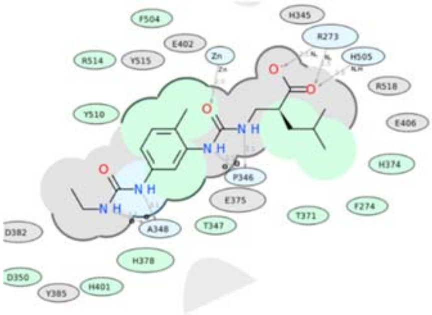	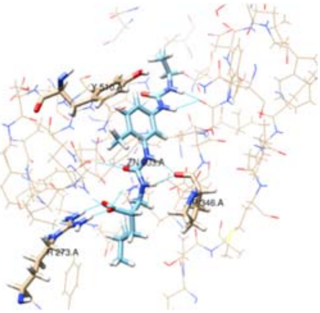

3	Z4422831009	-34.2	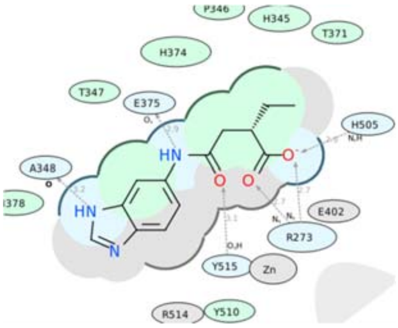	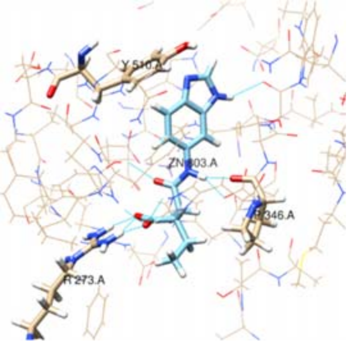
4	Z2899432355	-37.81	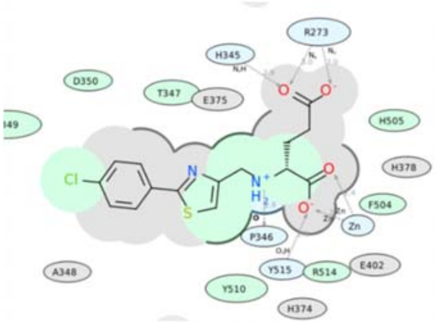	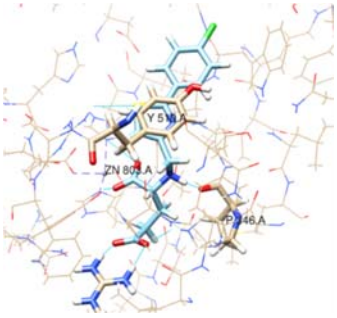
5	Z2793145343	-29.9	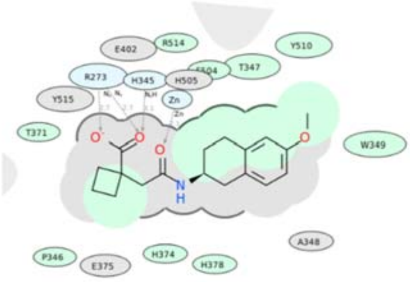	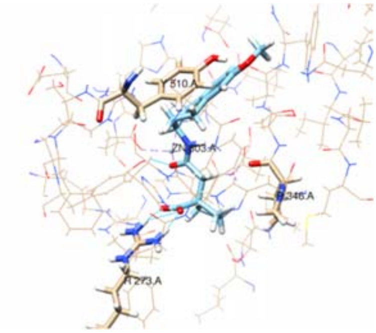
6	Z2396152156	-34.8	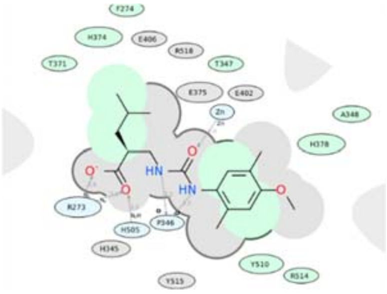	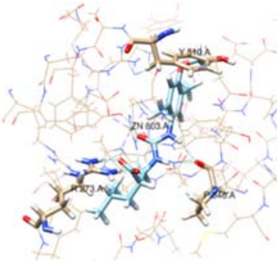

7	Z3510691032	-28.99	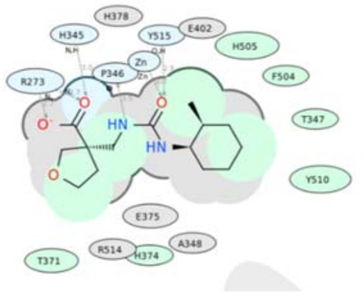	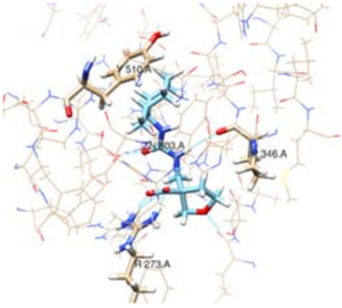
8	PV-002719568598	-30.33	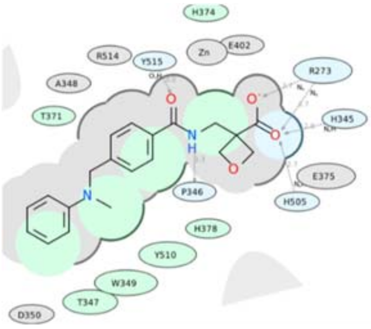	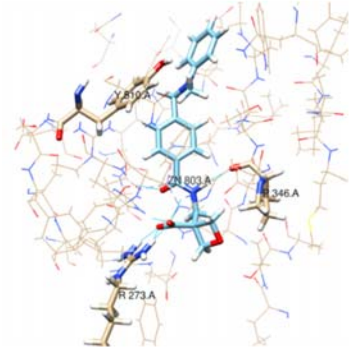
9	Z2610472638	-36.86	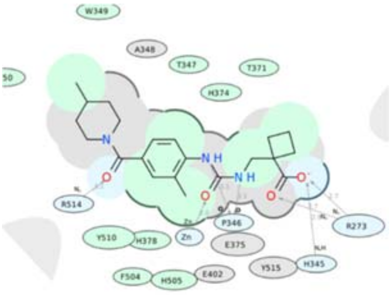	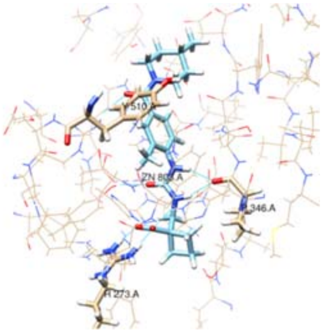
10	Z2596054371	-28.65	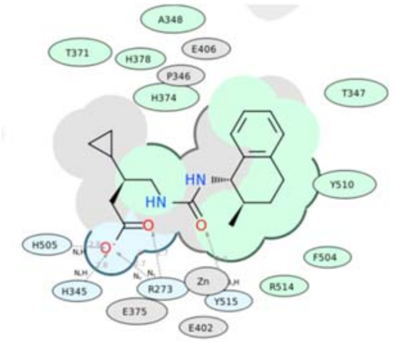	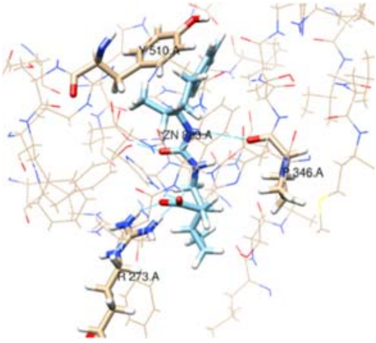

11	PV-002769423394	-39.26	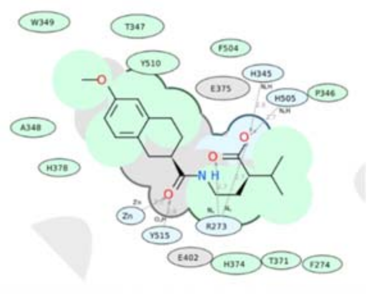	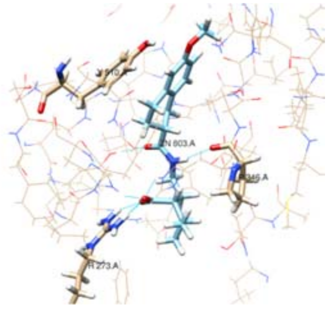
12	Z2596042648	-29.59	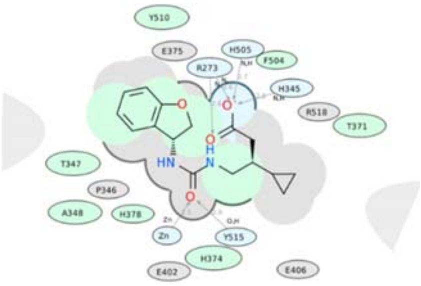	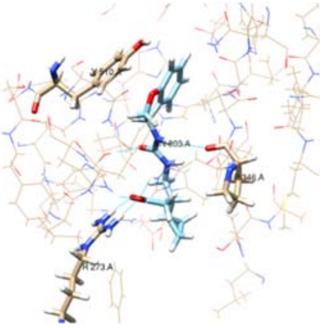
13	Z4422771675	-34.34	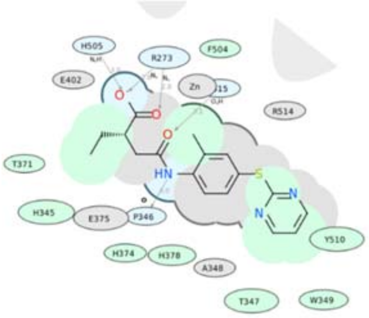	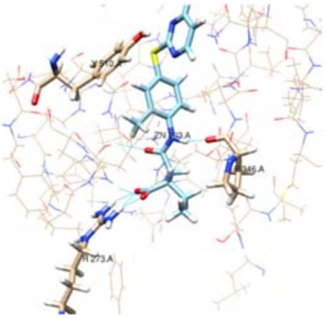
14	Z1213003608	-32.94	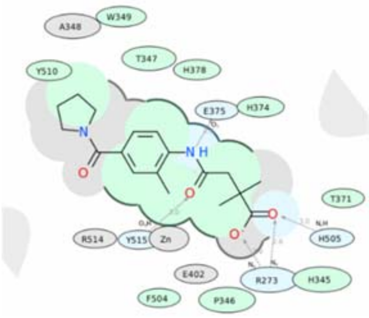	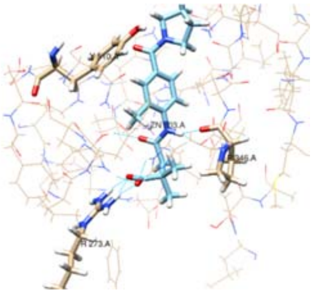

15	PV-002989187384	-34.78	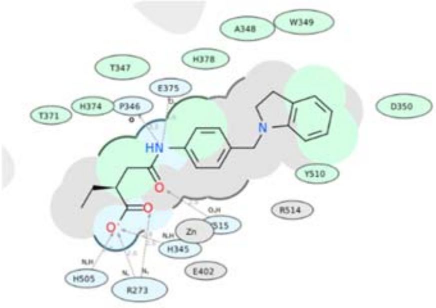	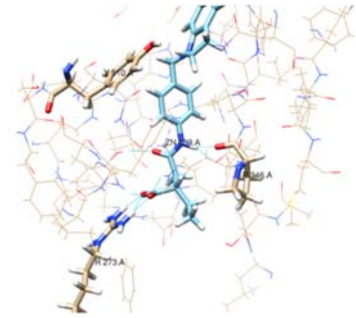
16	Z3654114554	-32.68	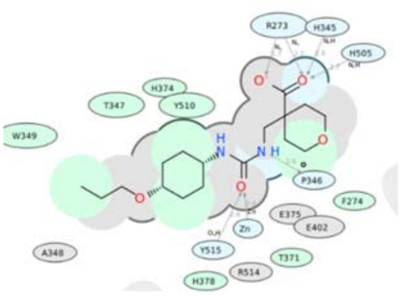	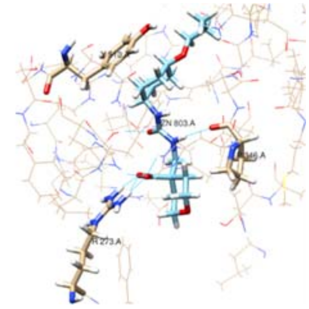
17	1Z2493059739	-38.68	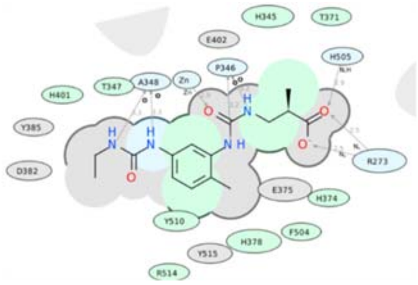	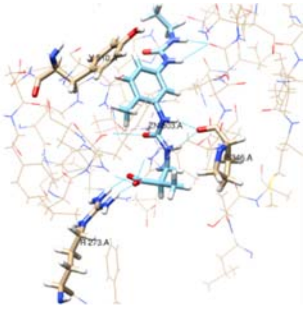
18	Z3510749222	-28.1	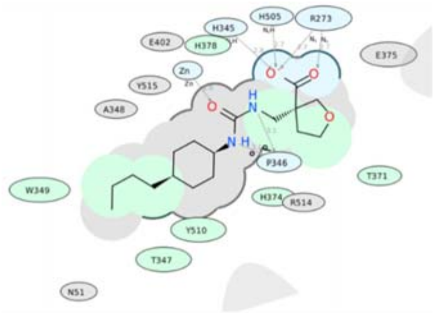	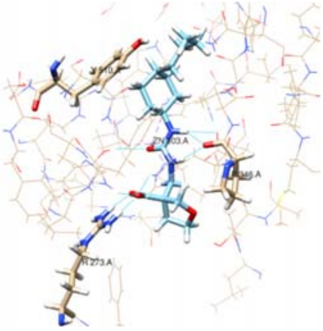

19	Z3390587460	-33.61	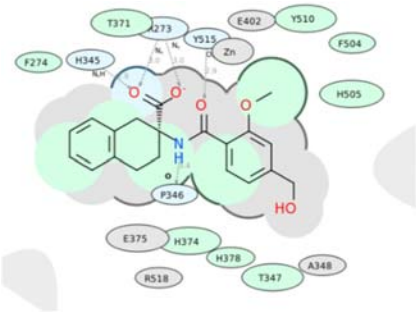	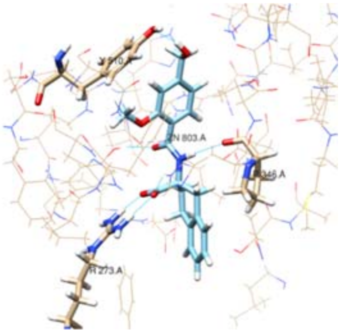
20	Z2362216591	-40.83	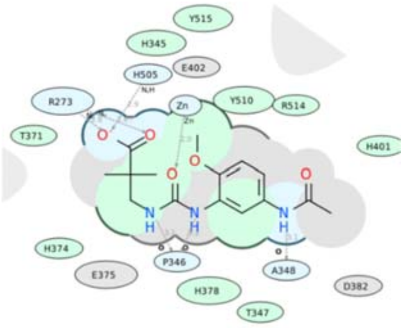	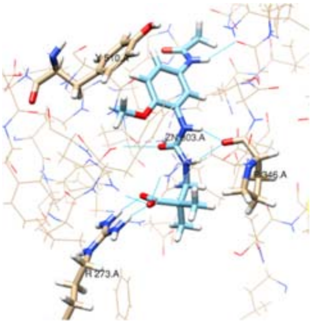
